# Dialysis Adequacy, Nutritional Target Attainment, and Intradialytic Amino Acid Loss in Maintenance Hemodialysis Patients: Implications for Clinical Management

**DOI:** 10.3390/healthcare14142186

**Published:** 2026-07-20

**Authors:** Shuo-Ming Hsu, Yu-Juei Hsu, Shiow-Ling Lee, Ming-Tse Lin

**Affiliations:** 1 Department of Chemical Engineering and Biotechnology, Tatung University, Taipei 104327, Taiwan; d10609001@ms.ttu.edu.tw (S.-M.H.); slee@gm.ttu.edu.tw (S.-L.L.); 2 Division of Nephrology, Tri-Service General Hospital Songshan Branch, Taipei 105011, Taiwan; yujuei@mail.ndmctsgh.edu.tw

**Keywords:** hemodialysis, dialysis adequacy, protein intake attainment, phosphorus, albumin, amino acid reduction, nutritional status

## Abstract

**Background**: Malnutrition and protein-energy wasting (PEW) are prevalent in maintenance hemodialysis (HD) patients and pose significant challenges to clinical care. Although adequate dietary protein intake is essential for maintaining nutritional status, phosphorus control often complicates nutritional management. In addition, HD is associated with dialysis-related amino acid (AA) loss, which may further aggravate nutritional vulnerability. This study examined the associations of dialysis adequacy with nutritional target attainment, biochemical parameters, and intradialytic AA reduction in maintenance HD patients. **Methods:** This cross-sectional study included 45 stable maintenance HD patients. Dietary intake was assessed using a 2-day pre-dialysis dietary record, and protein and energy intake attainment were calculated relative to recommended targets. Pre-HD and post-HD plasma AA concentrations were measured to determine intradialytic AA reduction. Correlation analyses, group comparisons, and multivariable regression analyses were performed. **Results:** Serum albumin was not significantly associated with protein intake, suggesting that it may not adequately reflect current dietary protein adequacy when used as an isolated marker. Patients with adequate protein intake attainment had lower serum phosphorus levels; however, after adjustment, dialysis adequacy assessed by spKt/V, rather than protein intake attainment itself, remained independently associated with serum phosphorus levels. Dialysis adequacy was significantly associated with both protein and energy intake attainment. HD was associated with significant reductions in most plasma AAs. Patients with adequate protein intake attainment showed lower intradialytic reductions in total amino acids, essential amino acids, branched-chain amino acids, and aromatic amino acids. **Conclusions:** Dialysis adequacy was associated with nutritional target attainment, phosphorus control, and intradialytic plasma AA reduction in maintenance HD patients. Adequate protein intake attainment was associated with lower category-specific AA reduction, whereas serum albumin alone did not reflect current dietary protein adequacy. These findings should be interpreted as associations and confirmed in larger prospective studies.

## 1. Introduction

Protein–energy wasting (PEW) refers to a pathological condition characterized by the loss of body protein and energy stores, including skeletal muscle and fat mass, in patients with chronic kidney disease. It is a prevalent and clinically significant complication in patients with end-stage kidney disease (ESKD) receiving maintenance hemodialysis (HD), and it is strongly associated with increased morbidity and mortality [1,2,3]. To counteract this catabolic state, clinical practice guidelines recommend achieving adequate dietary protein and energy intake to maintain metabolic homeostasis [4,5]. However, consistently meeting these nutritional targets remains a persistent challenge in routine healthcare delivery.

Multiple factors contribute to inadequate dietary intake, including uremic anorexia and restrictive dietary recommendations [6,7]. From a clinical management perspective, the dilemma between maintaining protein adequacy and controlling serum phosphorus levels is particularly acute. Concerns regarding hyperphosphatemia frequently lead to conservative dietary counseling that may unintentionally limit protein intake [8]. Recent evidence suggests that optimizing dialysis adequacy (spKt/V) may be an underrecognized facilitator of nutritional intake, as improved toxin clearance can alleviate uremic symptoms and support better dietary adherence [6,7].

Beyond diminished dietary intake, the HD procedure itself imposes recurring metabolic stress. Low-molecular-weight and water-soluble free amino acids (AAs) are readily cleared across the dialyzer membrane, resulting in obligatory intradialytic AA loss [9,10]. Over time, these recurrent losses exacerbate negative nitrogen balance and contribute to dialysis-related catabolism, further impairing overall nutritional status [11,12]. Mechanistic studies suggest that proactive nutrient provision may mitigate these processes; specifically, sufficient energy intake can exert a protein-sparing effect that attenuates endogenous proteolysis and preserves circulating AA pools [13,14,15]. Recognizing and managing this dialysis-induced metabolic shift is therefore a critical component of comprehensive patient care.

Despite the clinical importance of these interactions, real-world evidence integrating dialysis adequacy, nutritional target attainment, biochemical control, and intradialytic AA loss remains limited. Previous studies have largely focused on isolated aspects of HD nutrition, such as dialysis-related AA clearance or the efficacy of oral supplements. Relatively few investigations have jointly evaluated these interrelated factors within the same cohort [16,17,18,19,20]. Understanding these dynamics is essential for developing integrated, evidence-based nutritional protocols in HD populations.

Therefore, the present cross-sectional study examined the associations between dialysis adequacy (spKt/V), nutritional target attainment, biochemical parameters, and category-specific intradialytic AA loss in maintenance HD patients. We hypothesized that higher dialysis adequacy would be associated with better attainment of recommended protein and energy intake targets, as well as more favorable biochemical profiles. Furthermore, we hypothesized that better nutritional target attainment—particularly adequate protein intake—would be associated with lower intradialytic AA reduction during HD, thereby providing actionable insights for optimizing clinical management.

## 2. Materials and Methods

### 2.1. Step 1: Study Design, Setting, and Participant Selection

This observational cross-sectional study was conducted at a single center: the Hemodialysis Center of the Tri-Service General Hospital (Neihu Main Campus) in Taipei, Taiwan. Participants were enrolled using a convenience sampling method, recruiting directly from the attending physician’s primary hemodialysis patient pool. Eligible candidates included patients aged ≥20 years who had been undergoing thrice-weekly maintenance hemodialysis (HD) for at least 3 months. To minimize confounding factors, stable status was strictly required, defined as the absence of hospitalization, acute infection, active malignancy, or major changes in dialysis prescription during the preceding 3 months. Patients with severe comorbid conditions that could substantially confound nutritional or biochemical assessments were excluded.

**Figure 1:**
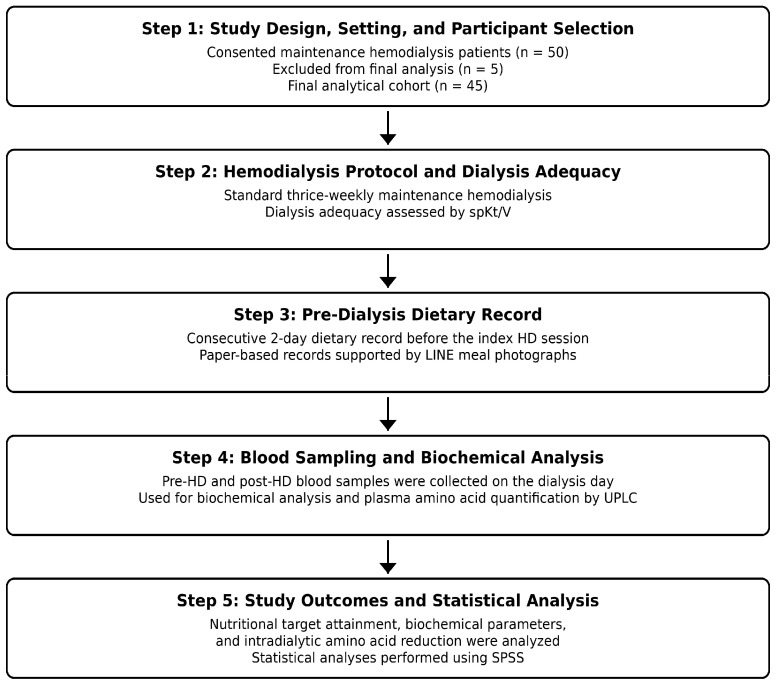
Study workflow of the present study. A total of 50 maintenance hemodialysis patients initially consented to participate. After exclusion of 5 patients, 45 patients were included in the final analysis.

The initial target for this exploratory physiological study was set at 50 participants. No formal a priori sample size calculation was performed because this was an exploratory observational study based on available patients from a single dialysis unit. During the study period, 5 patients were excluded from the final analysis due to mortality (n = 1), acute abnormal clinical status including severe calculi (n = 2), and incomplete 2-day dietary records (n = 2). Consequently, a final cohort of 45 stable maintenance HD patients (35 men and 10 women) was included in the analysis. The detailed participant selection process is illustrated in Figure 1. The study protocol was approved by the Institutional Review Board of Tri-Service General Hospital (TSGHIRB No. B202105030). Written informed consent was obtained from all participants prior to enrollment, in accordance with the Declaration of Helsinki.

### 2.2. Step 2: Hemodialysis Protocol and Dialysis Adequacy

All participants received standard thrice-weekly maintenance HD using bicarbonate-based dialysate. The standard dialysis prescriptions were as follows: session duration, 238.4 ± 14.5 min; blood flow rate (Qb), 272.3 ± 35.1 mL/min; dialysate flow rate (Qd), 500 mL/min; and ultrafiltration volume, 2.34 ± 0.96 kg/session. Dialyzer selection was determined by the treating nephrologists according to each patient’s dialysis prescription and clinical condition. Most patients were treated using two dialyzer models: TORAYLIGHT NV-21U dialyzer (Toray Medical Co., Ltd., Tokyo, Japan) and REXEED-18A dialyzer (Asahi Kasei Medical Co., Ltd., Tokyo, Japan). TORAYLIGHT NV-21U is a high-flux dialyzer with a HYDROLINK membrane and an effective surface area of 2.1 m^2^, whereas REXEED-18A is a high-flux dialyzer with a polysulfone membrane and an effective surface area of 1.8 m^2^. The remaining patients were treated with other dialyzer models according to individual clinical prescriptions. Dialysis adequacy was quantified as single-pool Kt/V for urea (spKt/V), calculated using the second-generation Daugirdas formula (single-pool, variable-volume model) [21]. The exact formula applied was spKt/V = −ln(R − 0.008 × t) + (4 − 3.5 × R) × (UF/W), where ln is the natural logarithm, R is the ratio of post-dialysis to pre-dialysis blood urea nitrogen (BUN), t is the dialysis session duration in hours, UF is the ultrafiltration volume in liters, and W is the post-dialysis body weight in kilograms. This calculation was automatically performed through the dialysis system’s dedicated clinical management software. The urea reduction ratio (URR) was also recorded as a supplementary indicator of dialysis efficiency.

### 2.3. Step 3: Nutritional Assessment and Stratification

Dietary intake was assessed using a consecutive 2-day dietary record during the interdialytic interval (specifically the two non-dialysis days strictly preceding the HD session to avoid acute treatment-related anorexia). The primary dietary data collection was paper-based. To enhance data accuracy and patient compliance, a dedicated Medical-Patient Health Education LINE Official Account was utilized as an interactive, two-way communication platform. Participants used this official group to conveniently submit real-time dietary photographs and meal times over the 2-day period. This platform enabled the research team to actively track patient progress and send instant reminders for completing their dietary records, allowing the certified clinical dietitian to objectively evaluate and verify portion sizes and nutritional intake using the Taiwan Food Exchange List [22]. Total dietary amino acid intake (AAI, g/kg/day) was calculated from protein-containing foods. Energy intake attainment (%) and protein intake attainment (%) were calculated using targets of 30 kcal/kg/day and 1.2 g/kg/day, respectively, based on the KDOQI 2020 Guidelines [4]. Patients were subsequently stratified into inadequate (<80% of target) and adequate (≥80% of target) protein intake attainment groups for comparative analysis. This prespecified 80% cutoff was conservatively set above the ≤75% intake threshold that indicates malnutrition risk according to the AND/ASPEN consensus [23].

### 2.4. Step 4: Blood Sampling and Biochemical Analysis

To evaluate intradialytic amino acid reduction, blood samples were collected directly from the patient’s vascular access site (arteriovenous fistula or graft) via the connected hemodialysis blood tubing. Patients were assessed in a non-fasting state to reflect real-world clinical conditions and to avoid disrupting their routine treatment schedules or nutritional habits. Sampling was strictly performed immediately before the initiation of hemodialysis (pre-HD) and within 5 min after the completion of the hemodialysis session (post-HD). To ensure strict standardization and avoid the confounding effects of access recirculation, post-HD samples were drawn using the recommended slow-flow/stop-pump technique by reducing the blood pump speed to approximately 50–100 mL/min for 15–30 s before sampling [24].

Importantly, to minimize patient burden and adhere to strict ethical standards, these procedures were performed strictly concurrently with the routine monthly clinical blood sampling required for standard dialysis adequacy monitoring. No additional venipunctures, extra blood volumes, or altered blood sampling schedules were imposed on the patients for the purpose of this study. Plasma proteins were precipitated using 10% (*w*/*v*) sulfosalicylic acid (SSA) [25]. Plasma amino acid (AA) concentrations were quantified using an ACQUITY UPLC H-Class System (Waters Corporation, Milford, MA, USA) after pre-column derivatization with 6-aminoquinolyl-N-hydroxysuccinimidyl carbamate (AQC) [26]. Amino acid analysis was performed using the AccQ-Tag Ultra C18 Amino Acid Analysis Kit (Waters Corporation, Milford, MA, USA) according to the manufacturer’s instructions [27]. AQC-derivatized amino acids were detected by ultraviolet absorbance at 260 nm [27,28]. Percentage reduction for each amino acid was calculated as (pre-HD − post-HD)/pre-HD × 100.

### 2.5. Step 5: Study Outcomes and Statistical Analysis

The primary outcomes were protein and energy intake attainment rates in relation to dialysis adequacy. Secondary outcomes included serum phosphorus, serum albumin, and category-specific intradialytic plasma amino acid reduction. Continuous variables are presented as mean ± standard deviation (SD). Data normality was assessed using the Shapiro–Wilk test. Group comparisons were performed using Welch’s *t*-test, and pre–post changes in plasma amino acid concentrations were evaluated using the Wilcoxon signed-rank test. Pearson correlation coefficients and multivariable linear regression analyses were utilized to assess associations among clinical parameters, adjusting for body mass index (BMI) where appropriate. All statistical analyses were performed using IBM SPSS Statistics for Windows, version 26.0 (IBM Corp., Armonk, NY, USA). A two-tailed *p* value < 0.05 considered statistically significant.

## 3. Results

### 3.1. Baseline Characteristics and Nutritional Profile

Baseline characteristics and clinical parameters are summarized in Table 1. The cohort had a mean age of 57.4 ± 10.5 years and a mean body mass index (BMI) of 23.9 ± 5.9 kg/m^2^. Dialysis adequacy, assessed by single-pool Kt/V (spKt/V), averaged 1.61 ± 0.13, and the mean ultrafiltration volume was 2.34 ± 0.96 kg per session. Mean daily energy and protein intake were 22.08 ± 7.14 kcal/kg/day and 1.03 ± 0.42 g/kg/day, corresponding to attainment rates of 73.61 ± 23.80% and 85.77 ± 35.37%, respectively. Baseline pre-dialysis serum albumin and phosphorus concentrations were 3.89 ± 0.29 g/dL and 5.13 ± 0.57 mg/dL, respectively.

### 3.2. Nutritional Attainment, Serum Albumin, and Phosphorus

**Figure 2:**
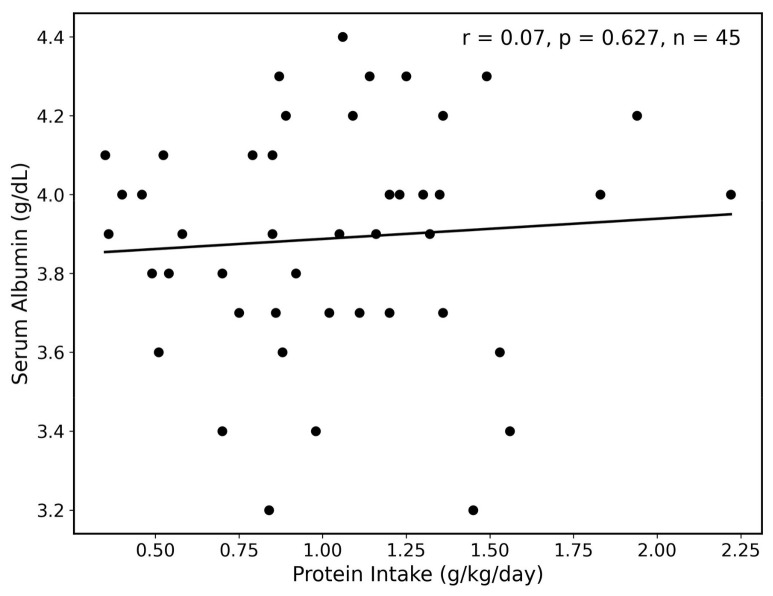
Association between protein intake (PI) and serum albumin (Alb) in maintenance hemodialysis patients. Scatter plot showing the relationship between PI (g/kg/day) and Alb (g/dL). The solid line represents the fitted linear regression line. Pearson correlation analysis showed no significant association (r = 0.074, *p* = 0.627; n = 45).

**Figure 3:**
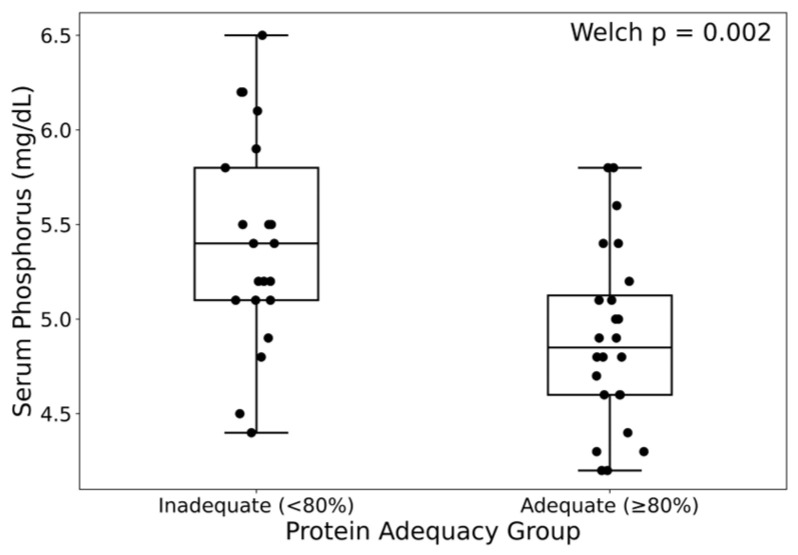
Serum phosphorus levels stratified by protein intake (PI) attainment groups. Box-and-whisker plot with individual data points overlaid comparing patients with PI attainment < 80% (n = 21) and ≥80% (n = 24). The boxes represent the interquartile range (IQR), the horizontal line within each box indicates the median, and the whiskers indicate the range. Group differences were assessed using Welch’s *t*-test.

Figure 2 shows no significant association between protein intake and serum albumin. Protein intake (g/kg/day) was not significantly correlated with serum albumin (r = 0.074, *p* = 0.627). When patients were stratified by protein intake attainment (<80% vs. ≥80%), the adequate intake group had significantly lower serum phosphorus levels than the inadequate intake group (4.90 ± 0.47 vs. 5.40 ± 0.56 mg/dL; *p* = 0.002) (Table 2). The distribution of serum phosphorus according to protein intake attainment group is shown in Figure 3.

However, in a multivariable linear regression model including the protein intake attainment group, spKt/V, and BMI, the protein intake attainment group was no longer independently associated with serum phosphorus levels (B = 0.052, *p* = 0.835). In contrast, higher spKt/V remained independently associated with lower serum phosphorus levels (B = −2.626, *p* = 0.011), whereas BMI was not independently associated with serum phosphorus (B = −0.007, *p* = 0.598). These findings suggest that the observed group difference in serum phosphorus was more strongly related to dialysis adequacy than to protein intake attainment itself.

### 3.3. Dialysis Adequacy and Nutritional Target Attainment

Patients in the adequate protein intake attainment group had significantly higher spKt/V values than those in the inadequate protein intake attainment group (1.71 ± 0.09 vs. 1.50 ± 0.06; *p* < 0.001; Table 2). To address potential body-size-related coupling between dialysis adequacy and weight-based nutritional attainment indices, additional analyses were performed using BMI as the adjusted covariate (Table 3). After BMI adjustment, the associations between spKt/V and protein intake attainment and between spKt/V and energy intake attainment remained significant (partial r = 0.936 and 0.715, respectively; both *p* < 0.001). In multivariable linear regression analyses, spKt/V remained independently associated with both protein intake attainment (B = 256.93, *p* < 0.001) and energy intake attainment (B = 112.21, *p* < 0.001). These findings indicate that better dialysis adequacy was closely associated with improved attainment of both protein and energy intake targets.

### 3.4. Pre- to Post-Dialysis Changes in Plasma Amino Acid Concentrations

Pre-HD and post-HD plasma amino acid concentrations and percentage reductions are summarized in Table 4. Aspartate was excluded from analysis because of analytical instability. Percentage reduction was calculated as (pre-HD − post-HD)/pre-HD × 100, and pre-HD and post-HD concentrations were compared using the Wilcoxon signed-rank test. Plasma concentrations of most measured amino acids decreased significantly after HD, supporting the presence of substantial intradialytic amino acid depletion. The largest percentage reductions were observed for cysteine (76.0%), histidine (52.5%), proline (46.5%), tyrosine (45.5%), alanine (41.6%), and arginine (41.5%). In contrast, tryptophan showed a slight, non-significant increase after HD.

### 3.5. Associations of Protein Intake Attainment with Category-Specific Intradialytic Plasma Amino Acid Reduction

**Figure 4:**
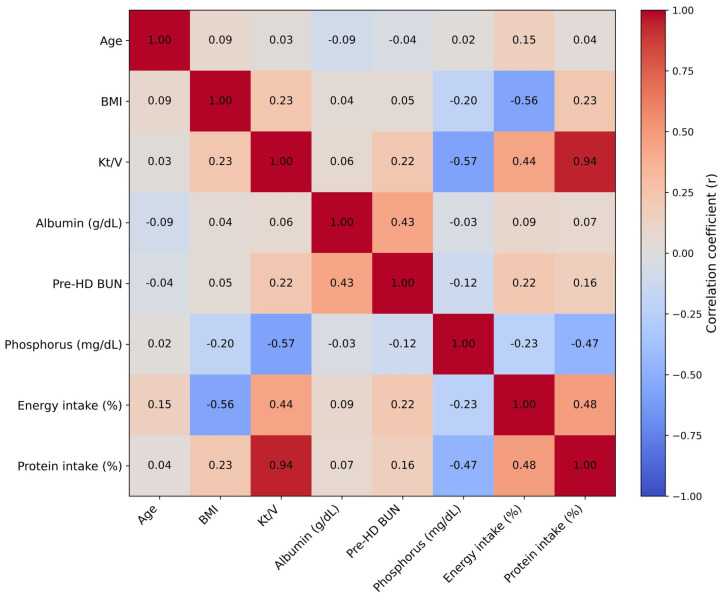
Correlation heatmap of clinical, biochemical, and nutritional variables. The values represent Pearson correlation coefficients (r). Color intensity reflects the strength and direction of the correlations. spKt/V: single-pool Kt/V; Alb: albumin; pre-BUN: pre-dialysis blood urea nitrogen; Phos: serum phosphorus; EI: energy intake attainment; PI: protein intake attainment.

**Figure 5:**
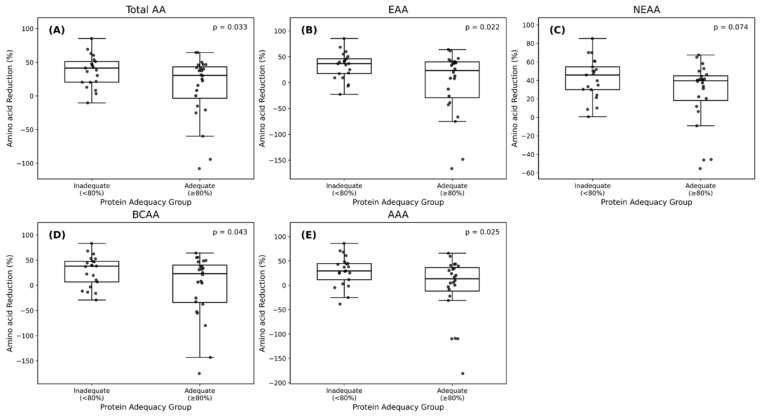
Comparisons of category-specific intradialytic plasma amino acid reduction according to protein intake attainment. Box-and-whisker plots with individual data points comparing patients with inadequate protein intake attainment (<80% of the recommended target) and adequate protein intake attainment (≥80% of the recommended target). Panel (**A**) shows total amino acids (Total AA), (**B**) essential amino acids (EAA), (**C**) non-essential amino acids (NEAA), (**D**) branched-chain amino acids (BCAA), and (**E**) aromatic amino acids (AAA). Boxes indicate the interquartile range, center lines indicate the median, and whiskers indicate the data range. Group differences were assessed using Welch’s *t*-test.

Correlation coefficients between category-specific intradialytic plasma amino acid reduction and clinical and nutritional variables are summarized in Table 5 and reflected in the overall correlation heatmap (Figure 4). Figure 5 compares category-specific intradialytic plasma amino acid reduction between patients with inadequate and adequate protein intake attainment. Patients in the adequate protein intake attainment group showed lower percentage reductions across several amino acid categories. Group differences were significant for total amino acids (*p* = 0.033), essential amino acids (*p* = 0.022), branched-chain amino acids (*p* = 0.043), and aromatic amino acids (*p* = 0.025), whereas non-essential amino acids showed a similar but non-significant trend (*p* = 0.074). These groupwise findings were generally consistent with the inverse correlations observed in Table 5, in which protein intake attainment was significantly inversely associated with aromatic amino acid reduction and showed similar inverse trends for the other amino acid categories.

## 4. Discussion

This study showed that dialysis adequacy was associated with nutritional target attainment and biochemical control in maintenance hemodialysis patients. A single HD session was associated with substantial reductions in circulating plasma amino acid (AA) concentrations, consistent with the recurring metabolic stress imposed by dialysis [9,10]. At the same time, patients with adequate protein intake attainment showed lower intradialytic AA reduction across multiple amino acid categories, particularly total amino acids, essential amino acids, branched-chain amino acids, and aromatic amino acids. Taken together, these findings suggest the interconnected nature of dialysis delivery, nutritional intake, and metabolic stress during HD, highlighting the need for comprehensive nutritional management in routine clinical care [9,10,12].

### 4.1. Intradialytic Amino Acid Loss as a Recurrent Catabolic Stressor in Clinical Care

The substantial post-dialysis reductions in plasma AA concentrations observed in this study are consistent with prior work showing that free AAs are readily removed during hemodialysis because of their low molecular weight and water solubility [9,10]. Classical studies quantified intradialytic free AA losses at approximately 6 to 8 g per session, whereas more recent reviews suggest that these losses may reach 10–12 g depending on dialyzer permeability [9,10]. In our cohort, the marked reductions in cysteine (76.0%) and histidine (52.5%), together with decreases in multiple non-essential and aromatic AAs, were consistent with this magnitude of depletion. Over time, this nontrivial nutrient clearance may contribute to negative nitrogen balance [9,10]. Mechanistic studies have further shown that the HD procedure itself can increase skeletal muscle protein breakdown by approximately 20% compared with non-dialysis days, thereby exacerbating net protein loss [11,12]. Collectively, these findings are consistent with intradialytic nutrient loss as a recurrent catabolic stressor that healthcare providers must actively account for to reduce patient vulnerability to PEW [1,2,3]. Recent crossover data further suggest that dialysis-related amino acid removal can be at least partially compensated by targeted protein ingestion [29].

### 4.2. Protein Intake Attainment as a Tangible Clinical Benchmark

An important finding of this study was that patients with adequate protein intake attainment showed lower intradialytic AA reduction across multiple amino acid categories. In groupwise analyses, significant differences were observed for total amino acids, essential amino acids, branched-chain amino acids, and aromatic amino acids, whereas non-essential amino acids showed a similar but non-significant trend. These findings suggest that achieving at least 80% of the recommended protein intake target serves as a practical clinical benchmark associated with a lower degree of intradialytic amino acid depletion.

This pattern is biologically plausible. Adequate protein intake may help maintain circulating amino acid availability and support metabolic resilience under the recurrent catabolic stress of hemodialysis. Although the present cross-sectional design does not permit causal inference, the observed groupwise differences are consistent with the concept that proactively meeting nutritional targets may partially buffer dialysis-associated amino acid depletion. The finding that aromatic amino acid reduction also showed an inverse correlation with protein intake attainment (Table 5) further supports this interpretation within a clinical context.

### 4.3. Managing the Phosphorus “Paradox” in Routine Healthcare

A major clinical concern is that encouraging protein intake may worsen serum phosphorus, particularly when dietary phosphorus density is high or the phosphorus-to-protein ratio is unfavorable [8,30,31,32]. Noori et al. reported that a dietary phosphorus-to-protein ratio exceeding 16 mg/g is independently associated with a stepwise increase in mortality [8]. In contrast, our unadjusted data showed that the inadequate protein intake group had higher serum phosphorus concentrations (5.40 vs. 4.90 mg/dL), suggesting that restricting protein intake alone does not guarantee phosphorus control [8,30,31,32]. However, this association was attenuated after adjustment for spKt/V and BMI, suggesting that dialysis adequacy rather than protein intake attainment per se was the dominant determinant. Specifically, higher spKt/V remained independently associated with lower serum phosphorus levels. These findings suggest that the lower phosphorus levels observed in the adequate intake group were primarily explained by dialysis adequacy. Accordingly, our results support a more integrated approach in which phosphorus management is combined with adequate dialysis delivery and structured dietary education—such as the practical application of food exchange lists to guide high-quality protein choices—rather than indiscriminate protein restriction [4,30,31,32].

### 4.4. Kt/V as a Clinically Relevant Indicator in Nutritional Management

The strong BMI-adjusted association between spKt/V and protein intake attainment should be interpreted with caution. Both spKt/V and weight-normalized nutritional attainment indices are influenced by body size, and therefore mathematical coupling related to shared body-size normalization cannot be fully excluded. Although BMI-adjusted analyses were performed to partially address this concern, residual coupling may still have contributed to the magnitude of the observed association. From a biological perspective, higher delivered dialysis dose may also be clinically linked to improved metabolic control and reduced uremic symptom burden, including anorexia, thereby facilitating better dietary adherence [6,7]. Therefore, the observed association between spKt/V and nutritional target attainment should be interpreted as potentially reflecting both physiological relevance and methodological coupling, and it warrants confirmation in larger prospective studies using additional non-weight-normalized nutritional measures.

### 4.5. Overcoming the Limitations of Single Nutritional Biomarkers

The absence of a meaningful association between protein intake and serum albumin (r = 0.074) is consistent with the literature emphasizing the multifactorial determinants of albumin in ESKD [33,34,35]. Classic studies showed that serum albumin synthesis is strongly influenced by systemic inflammation, such that albumin may remain low despite adequate dietary protein intake [33,35]. While serum albumin remains prognostically valuable, it may not sensitively reflect short-term dietary changes [33,34,35,36]. Accordingly, nutritional attainment indices, interpreted alongside objective metabolic measures such as AA profiles, equip healthcare teams with a more precise and comprehensive assessment of current dietary performance under dialysis-related catabolic stress [4,9,10].

### 4.6. Limitations

This study has several limitations. First, its cross-sectional design precludes causal inference. Therefore, the observed associations between dialysis adequacy, nutritional target attainment, phosphorus control, and amino acid reduction should be interpreted as hypothesis-generating rather than causal. Second, the modest sample size may have limited statistical power, particularly for multivariable analyses, and may reduce the robustness and generalizability of the findings. No formal a priori power calculation was performed because this was an exploratory observational study based on eligible patients from a single dialysis unit. Third, potentially important confounders, including inflammatory markers such as C-reactive protein, residual kidney function, diabetes status, comorbidity burden, dialysis vintage, phosphate binder adherence, and body composition, were not fully available for adjustment. These factors may influence nutritional status, serum phosphorus and albumin concentrations, and amino acid metabolism; therefore, residual confounding cannot be excluded. In addition, because both spKt/V and weight-normalized nutritional attainment indices are influenced by body size, mathematical coupling may have contributed to the strong observed association between dialysis adequacy and protein intake attainment, even after BMI adjustment. Fourth, dietary intake was assessed using a 2-day dietary record strictly preceding the hemodialysis session. Although this approach was intended to capture the interdialytic period and reduce the influence of acute post-dialysis symptoms, it may not represent long-term habitual dietary intake. Therefore, the reported attainment rates should be interpreted as short-term intake indicators rather than definitive measures of habitual intake. Finally, plasma amino acid concentrations reflect circulating amino acid availability before and after a single dialysis session, but they do not directly measure total amino acid loss, whole-body protein turnover, muscle mass preservation, or long-term nutritional outcomes.

### 4.7. Clinical Implications

Collectively, these findings may support an integrated management strategy for maintenance HD care: optimizing dialysis adequacy, ensuring adequate nutrient intake, and managing phosphorus through dietary quality and dialysis delivery rather than indiscriminate protein restriction [4,8,30,31,32]. For healthcare systems, routinely tracking attainment rates—rather than just prescribed targets—may help identify patients at elevated risk for ongoing catabolic stress. This proactive approach allows for early intervention by multidisciplinary teams, potentially reducing the long-term clinical and economic burden associated with protein-energy wasting in the dialysis population [37,38].

## 5. Conclusions

In maintenance hemodialysis patients, dialysis adequacy (spKt/V) was associated with protein intake attainment and phosphorus control, while serum albumin was not significantly associated with current dietary protein intake. Adequate protein intake attainment was also associated with lower category-specific intradialytic plasma amino acid reduction. These findings suggest that dialysis adequacy, dietary target attainment, phosphorus management, and dialysis-associated amino acid depletion are clinically interrelated. However, given the cross-sectional design and modest sample size, these findings should be interpreted as associations rather than causal evidence and should be confirmed in larger prospective studies.

## Figures and Tables

**Table 1:** Baseline characteristics, nutritional intake, and clinical parameters of maintenance hemodialysis patients (N = 45).

Parameters	Values
Demographics	
Sex, male, n (%)	35 (77.8)
Sex, female, n (%)	10 (22.2)
Age (years)	57.4 ± 10.5
BMI (kg/m^2^)	23.9 ± 5.9
Dialysis parameters	
spKt/V	1.61 ± 0.13
UF volume (kg/session)	2.34 ± 0.96
Nutritional intake assessment	
EI (kcal/kg/day)	22.08 ± 7.14
EI attainment (%)	73.61 ± 23.80
PI (g/kg/day)	1.03 ± 0.42
PI attainment (%)	85.77 ± 35.37
Baseline biochemical parameters (pre-HD)	
Alb (g/dL)	3.89 ± 0.29
BUN (mg/dL)	68.62 ± 17.06
Cr (mg/dL)	10.81 ± 1.97
Phos (mg/dL)	5.13 ± 0.57
Ca (mg/dL)	9.13 ± 0.88

Data are presented as mean ± standard deviation (SD) unless otherwise indicated. Baseline biochemical parameters were measured immediately before the hemodialysis session (pre-HD). BMI, body mass index; spKt/V, single-pool Kt/V; UF, ultrafiltration; EI, energy intake; PI, protein intake; Alb, serum albumin; BUN, blood urea nitrogen; Cr, serum creatinine; Phos, serum phosphorus; Ca, total calcium.

**Table 2:** Comparison of clinical, nutritional, and biochemical characteristics stratified by protein intake attainment.

Variable	Inadequate PI (<80%) (n = 21)	Adequate PI (≥80%) (n = 24)	*p* Value
Demographics			
Sex (male/female), n	17/4	18/6	—
Age (years)	58.90 ± 8.35	56.00 ± 12.09	0.349
BMI (kg/m^2^)	22.78 ± 6.31	24.83 ± 5.48	0.255
Nutritional assessment			
EI (kcal/kg/day)	19.26 ± 6.78	24.55 ± 6.63	0.012 *
PI (g/kg/day)	0.67 ± 0.20	1.34 ± 0.30	<0.001 *
AAI (g/kg/day)	0.69 ± 0.25	1.20 ± 0.40	<0.001 *
Dialysis & Biochemical			
spKt/V	1.50 ± 0.06	1.71 ± 0.09	<0.001 *
Alb (g/dL)	3.86 ± 0.27	3.92 ± 0.32	0.497
pre-BUN (mg/dL)	67.52 ± 16.57	69.58 ± 17.77	0.690
Phos (mg/dL)	5.40 ± 0.56	4.90 ± 0.47	0.002 *

Values are presented as mean ± standard deviation (SD) unless otherwise indicated. BMI: body mass index; EI: energy intake; PI: protein intake; AAI: amino acid intake; spKt/V: single-pool Kt/V; Alb: albumin; pre-BUN: pre-dialysis blood urea nitrogen; Phos: serum phosphorus. Patients were stratified by PI attainment (<80% vs. ≥80% of the target intake of 1.2 g/kg/day). * *p* < 0.05 indicates statistical significance.

**Table 3:** BMI-adjusted analyses of the associations between dialysis adequacy and nutritional attainment.

Analysis	Outcome	Predictor	Covariate	Coefficient	*p* Value
Partial correlation	PI attainment (%)	spKt/V	BMI	0.936	<0.001
Partial correlation	EI attainment (%)	spKt/V	BMI	0.715	<0.001
Linear regression	PI attainment (%)	spKt/V	BMI	256.93	<0.001
Linear regression	EI attainment (%)	spKt/V	BMI	112.21	<0.001

BMI-adjusted analyses were performed to address potential body-size-related coupling. Partial correlation coefficients are reported as adjusted r values. Regression coefficients are unstandardized coefficients (B). BMI: body mass index (kg/m^2^); PI: protein intake attainment; EI: energy intake attainment; spKt/V: single-pool Kt/V.

**Table 4:** Pre-HD and post-HD plasma amino acid concentrations and percentage reductions during hemodialysis.

Amino Acid	Pre-HD (μmol/L)	Post-HD (μmol/L)	Reduction (%)	*p* Value
Cysteine (Cys)	111.86 ± 47.41	26.88 ± 10.34	76.0	<0.001 *
Histidine (His)	51.88 ± 58.96	24.65 ± 22.45	52.5	<0.001 *
Proline (Pro)	312.70 ± 129.01	167.21 ± 80.74	46.5	<0.001 *
Tyrosine (Tyr)	57.59 ± 31.80	31.37 ± 17.41	45.5	<0.001 *
Alanine (Ala)	289.68 ± 83.49	169.26 ± 57.76	41.6	<0.001 *
Arginine (Arg)	108.12 ± 39.09	63.22 ± 31.41	41.5	<0.001 *
Asparagine (Asn)	103.61 ± 26.67	64.65 ± 17.66	37.6	<0.001 *
Valine (Val)	192.62 ± 55.18	126.82 ± 53.81	34.2	<0.001 *
Phenylalanine (Phe)	89.69 ± 26.45	60.40 ± 20.01	32.7	<0.001 *
Threonine (Thr)	141.46 ± 49.81	97.15 ± 40.63	31.3	<0.001 *
Serine (Ser)	110.62 ± 32.62	79.71 ± 32.97	27.9	<0.001 *
Glutamic acid (Glu)	163.14 ± 49.11	118.50 ± 46.96	27.4	<0.001 *
Glycine (Gly)	282.25 ± 90.01	205.24 ± 64.68	27.3	<0.001 *
Lysine (Lys)	142.69 ± 42.98	104.86 ± 52.49	26.5	<0.001 *
Methionine (Met)	26.95 ± 9.59	20.10 ± 10.78	25.4	<0.001 *
Glutamine (Gln)	627.00 ± 146.40	486.62 ± 134.33	22.4	<0.001 *
Isoleucine (Ile)	68.34 ± 21.20	56.01 ± 28.02	18.0	0.015 *
Leucine (Leu)	104.35 ± 34.39	92.89 ± 43.40	11.0	0.118
Tryptophan (Trp)	26.75 ± 10.61	27.95 ± 9.82	−4.5	0.575
Aspartate (Asp)	14.53 ± 3.40	N/A	N/A	N/A

Data are presented as mean ± standard deviation (SD). Percentage reduction was calculated as (pre-HD − post-HD)/pre-HD × 100. Aspartate was excluded from analysis because of analytical instability. Pre-HD and post-HD concentrations were compared using the Wilcoxon signed-rank test. * *p* < 0.05.

**Table 5:** Pearson correlations between intradialytic plasma amino acid percentage reduction and clinical/nutritional variables.

Amino Acid Percentage	spKt/V	URR (%)	Alb (g/dL)	EI (%)	PI (%)	Pre-BUN (mg/dL)
Total AA	−0.282 (0.061)	−0.122 (0.425)	−0.089 (0.561)	−0.255 (0.091)	−0.285 (0.058)	−0.072 (0.638)
EAA	−0.290 (0.053)	−0.158 (0.300)	−0.056 (0.715)	−0.292 (0.052)	−0.292 (0.052)	−0.062 (0.686)
NEAA	−0.257 (0.088)	−0.059 (0.700)	−0.135 (0.377)	−0.184 (0.226)	−0.260 (0.085)	−0.086 (0.574)
BCAA	−0.251 (0.096)	−0.175 (0.250)	0.021 (0.891)	−0.266 (0.077)	−0.246 (0.103)	−0.044 (0.774)
AAA	−0.326 (0.029) *	−0.219 (0.148)	−0.089 (0.561)	−0.336 (0.024) *	−0.319 (0.033) *	−0.042 (0.784)

Values are presented as Pearson correlation coefficients (r), with exact two-tailed *p* values in parentheses. Category-specific amino acid reduction was calculated as the mean percentage reduction across amino acids within each predefined category. * *p* < 0.05 indicates statistical significance. Total AA: total amino acids; EAA: essential amino acids; NEAA: non-essential amino acids; BCAA: branched-chain amino acids; AAA: aromatic amino acids; spKt/V: single-pool Kt/V; URR: urea reduction ratio; Alb: albumin; EI: energy intake attainment; PI: protein intake attainment; pre-BUN: pre-dialysis blood urea nitrogen.

## Data Availability

The data presented in this study are available on request from the corresponding author. The data are not publicly available due to privacy and ethical restrictions.

## Abbreviations

The following abbreviations are used in this manuscript:

Abbreviation	Full Term
AA	Amino acid
AAA	Aromatic amino acid
AAI	Amino acid intake
Alb	Serum albumin
AQC	6-Aminoquinolyl-N-hydroxysuccinimidyl carbamate
BCAA	Branched-chain amino acid
BMI	Body mass index
BUN	Blood urea nitrogen
EAA	Essential amino acid
EI	Energy intake
ESKD	End-stage kidney disease
HD	Hemodialysis
KDOQI	Kidney Disease Outcomes Quality Initiative
NEAA	Non-essential amino acid
PEW	Protein–energy wasting
PI	Protein intake
spKt/V	Single-pool Kt/V
TSGH	Tri-Service General Hospital
UPLC	Ultra-performance liquid chromatography
URR	Urea reduction ratio
